# Machine learning-guided clinical pharmacist interventions improve treatment outcomes in tuberculosis patients: a precision medicine approach

**DOI:** 10.3389/frai.2025.1679837

**Published:** 2025-12-18

**Authors:** Dang Yi, Huanqing Liu, Qian Lei, Tingting Li

**Affiliations:** 1 Department of Clinical Research, Xianyang Hospital of Yan'an University, Xianyang, Shaanxi, China; 2 Information Management Office, Northwestern Polytechnical University, Xi’an, Shaanxi, China; 3 Department of Pharmacy, Xi’an Chest Hospital, Xi’an, Shaanxi, China

**Keywords:** precision medicine, tuberculosis, clinical pharmacist, machine learning, therapeutic drug monitoring

## Abstract

**Background:**

The heterogeneity in tuberculosis (TB) treatment responses necessitates a precision medicine approach. This study employed machine learning techniques to identify patient subtypes and optimize clinical pharmacist interventions.

**Methods:**

We conducted a prospective cohort study involving 467 TB patients (218 in the intervention group receiving machine learning-guided pharmacist care and 249 in the control group receiving standard care). Primary outcomes included time to sputum conversion (smear, culture, TB-RNA) and duration of hospitalization; secondary outcomes encompassed adverse event rates (hepatotoxicity, renal impairment, etc.), cost-effectiveness, and biomarker dynamics. Patient stratification was performed using unsupervised learning (k-means/PCA) on clinical and laboratory parameters. Treatment outcomes were assessed via Kaplan–Meier survival analysis and Cox proportional hazards modeling, with prespecified subgroup analyses by risk clusters. *Post hoc* analyses (e.g., correlation heatmaps of biomarkers) were explicitly labeled as exploratory. Cost-effectiveness was evaluated using incremental cost per quality-adjusted hospital day saved (ICER).

**Results:**

Machine learning identified 2 distinct patient subtypes (inflammatory vs. immunologic profiles). The intervention group showed significantly shorter hospital stays (primary outcome: median 49.0 vs. 57.0 days; log-rank *p* = 0.040). Adverse event rates were lower in the intervention group (26.1% vs. 27.7%). Cost analysis demonstrated potential savings of 5,000 CNY per patient in the intervention group. Limitations: Single-center design and modest sample size may limit generalizability. Unmeasured confounders (e.g., socioeconomic factors) could influence outcomes. *Post hoc* biomarker correlations require validation in independent cohorts.

**Conclusion:**

Machine learning-guided pharmacist interventions improved TB treatment outcomes and reduced costs. Future multicenter studies should validate subtype-specific benefits.

**Clinical trial registration:**

https://www.chictr.org.cn/ identifier ChiCTR2300074328.

## Highlights

This study demonstrated that machine learning-guided clinical pharmacist interventions improved tuberculosis treatment outcomes by enabling personalized care. Patient stratification and biomarker monitoring reduced hospital stays by 14% and lowered costs, highlighting the value of precision medicine in TB management.

## Introduction

Tuberculosis (TB) has resurged as the leading global cause of death from a single infectious agent post-COVID-19 pandemic, claiming approximately 1.25 million lives in 2023, initial treatment and cure remain key strategies for the prevention and control of tuberculosis (World health organization, 2024). Therapeutic drug monitoring (TDM) is a clinically valuable approach that can be utilized to optimize the treatment process of tuberculosis, as it enables the precise determination of drug concentration levels during the crucial initial treatment stage (Alffenaar et al., 2020). Clinical pharmacist interventions have demonstrated potential in improving medication adherence and reducing adverse events in TB care (Awad et al., 2024). However, traditional “one-size-fits-all” approaches fail to address fundamental challenges, such as patient heterogeneity in disease progression and treatment response (Cadena et al., 2017), complex interactions between clinical parameters and therapeutic outcomes (Cicchese et al., 2018), economic constraints requiring cost-effective personalized strategies (Dowdy et al., 2014), etc.

Machine learning (ML) offers transformative potential for addressing these limitations. Recent advances enable data-driven patient stratification using multidimensional clinical and laboratory biomarkers, facilitating targeted interventions (Meserve et al., 2025). Emerging evidence suggests ML models can predict TB treatment outcomes with >80% accuracy by integrating inflammatory markers, hepatic function indices, and immunological profiles (Mann et al., 2021).

Our study bridges two innovative domains: ML-guided patient stratification and precision clinical pharmacy. While prior research established the value of pharmacist interventions in TB (Khan et al., 2023), none have integrated real-time ML analytics to personalize these services based on biomarker-defined patient subtypes. This gap is critical given that 15–25% of TB patients experience treatment complications requiring individualized management (Nahid et al., 2016). This study filled this gap: By developing a machine learning framework (k-means/PCA), 467 tuberculosis patients were classified into two subtypes based on inflammatory, liver and immunological characteristics using biomarkers; at the same time, pharmacist intervention measures targeting the subtypes were implemented, including treatment drug monitoring and prevention of adverse events, verifying the effectiveness of a clinically feasible precision pharmacy model for tuberculosis management.

## Object and methods

### Study design and participants

We conducted a prospective cohort study at Xi’an Chest Hospital between September 2023 to and December 2024. The first participant was enrolled on September 15, 2023, and the last participant was enrolled on December 10, 2024. The study protocol was approved by the Ethics Committee for Medical Scientific Research of Xi’an Chest hospital (approval number: S2023-0002).

### Study population eligibility criteria

Inclusion criteria: (1) Patients diagnosed with pulmonary tuberculosis according to standardized diagnostic criteria (Khan et al., 2023); (2) *Mycobacterium tuberculosis*-positive as confirmed by smear, culture, or molecular methods (GeneXpert MTB/RIF, DNA/RNA amplification, melting curve analysis, etc.); (3) no prior anti-tuberculosis treatment; and (4) undergoing intensive-phase first-line anti-TB therapy.

Exclusion criteria: (1) Pregnancy; (2) age < 18 years; (3) severe hepatic impairment (Child-Pugh class C); (4) severe renal impairment (glomerular filtration rate < 30 mL/min); and (5) other severe baseline abnormalities.

Withdrawal criteria: (1) Treatment regimen modification; and (2) loss to follow-up.

### Clinical pharmacist intervention

To mitigate potential selection bias, participants were systematically recruited from three tuberculosis wards with comparable patient demographics and treatment protocols at Xi’an Chest Hospital.

Intervention group (pharmacist-led intervention group):

(1) Patients received an initial oral anti-tuberculosis regimen prescribed by physicians based on body weight: isoniazid (300 mg once daily for patients <50 kg, 400 mg once daily for patients ≥50 kg), rifampin (450 mg once daily for patients <50 kg, 600 mg once daily for patients ≥50 kg), pyrazinamide (15–30 mg/kg once daily), and ethambutol (15 mg/kg once daily).
(2) During the steady-state phase (after at least 1 week of treatment), nurses collected blood samples following a standardized operating procedure (SOP), which consisted of three key steps: ① On the evening before TDM, patients were provided with detailed instructions regarding medication administration and the next day’s blood sampling schedule, with specific emphasis on fasting for one hour before and after medication intake; ② on the morning of TDM, patients were reminded to take their medications at the predetermined time, followed by timed blood collection according to the protocol; and ③ blood samples were transported to the laboratory within 30 min after collection.
(3) Plasma concentrations of anti-tuberculosis drugs were quantified by laboratory technicians using liquid chromatography–tandem mass spectrometry (LC–MS/MS).
(4) Clinical pharmacists provided comprehensive pharmaceutical care throughout the treatment course, encompassing the following responsibilities: ① Assessing medication appropriateness; ② delivering medication education and counseling to patients; ③ monitoring therapeutic efficacy and adverse drug reactions; ④ providing TDM recommendations to physicians, including monitoring strategies and dosage adjustments; and⑤ advising nurses on optimal medication administration and blood sampling timing. The reference ranges for plasma concentrations were established based on previously reported.

Control group:

(1) Patients received the same initial oral anti-tuberculosis regimen as the intervention group.
(2) Blood samples were collected by nurses following routine procedures.
(3) Laboratory plasma concentration monitoring was consistent with the intervention group.
(4) Clinical pharmacists were only involved in observation and follow-up, with no treatment intervention or recommendations provided.

If the dosage exceeded the labeled range, patients provided written informed consent for off-label use in advance. The clinical team performed close monitoring and took immediate action (e.g., drug discontinuation or symptomatic management) for any adverse events, which were systematically documented and reported.

### Pharmacist intervention details

(1) Standardized pharmaceutical care (for all intervention group patients)

Standardized pharmaceutical care (for all intervention group patients):

The intervention group received a structured, pharmacist-integrated therapeutic drug monitoring service, which included the following components: Pre-medication monitoring consultation: The pharmacist would conduct a comprehensive drug assessment, including evaluating comorbidities, concurrent medications (such as liver protectants like compound glycyrrhizin), and potential drug interactions. Education on the importance of adhering to medication was provided to the patients, including optimal sampling times (such as 2 h after isoniazid/rifampicin) and monitoring of adverse reactions (such as liver toxicity symptoms). Individualized dose adjustment: Blood samples of isoniazid, rifampicin, pyrazinamide, and ethambutol were collected 5 days or more after the steady state of treatment. The pharmacist interpreted the therapeutic drug monitoring results based on the institution-specific therapeutic range (such as isoniazid C ~ 2 h~: 3–6 milligrams per liter) and adjusted the dose according to pharmacokinetic principles (such as the linear kinetics of isoniazid). For levels below the therapeutic threshold, the dose would be gradually increased, and therapeutic drug monitoring would be repeated 3 days later. Active pharmacovigilance: Laboratory test results (alanine aminotransferase, aspartate aminotransferase, bilirubin, creatinine) and symptoms (nausea, rash, joint pain) were monitored weekly. For cases of liver toxicity (alanine aminotransferase above three times the upper limit of normal), the pharmacist recommended using liver-protecting drugs (such as bicyclol) or suspending treatment, and then retesting according to the adjusted protocol. Multidisciplinary collaboration: The pharmacist communicated the dose adjustment and monitoring plan to the doctor through electronic health record alerts and weekly team meetings. Nurses received training on standardized blood collection protocols to minimize variations.

(2) Subgroup-specific intervention (based on machine learning stratification)

① Inflammation-dominant subgroup (CRP > 10 mg/L, elevated ALT/AST) Intensive monitoring: Conduct CRP and ALT/AST tests twice a week. Increase the frequency of blood drug concentration monitoring to once a week (as metabolism may be affected by inflammation). Intervention measures: Preventive liver protection treatment (such as using compound glycyrrhizin when ALT > 1.5 × ULN). Non-steroidal anti-inflammatory drugs (such as ibuprofen) are used for those with persistently elevated CRP.
② Immune dysregulation subgroup (CD4/CD8 < 1.5, low lymphocyte count) Intensive monitoring: Test lymphocyte subsets (CD4/CD8 ratio) every two weeks. Assess nutritional status (albumin, prealbumin). Intervention measures: Immune nutritional support (such as high-protein diet, vitamin D supplementation).

### Definition of ML-guided pharmacist care

ML-guided pharmacist care is defined as a systematic, data-driven approach that integrates machine learning risk stratification with evidence-based clinical decision support to deliver personalized pharmaceutical interventions. This approach differs from traditional pharmacist care in three key aspects: (1) predictive risk assessment using machine learning algorithms to identify high-risk patients, (2) personalized intervention protocols based on individual patient characteristics and predicted outcomes, and (3) continuous monitoring and adjustment of care plans based on real-time clinical data and biomarker responses.

### ML-guided care workflow

**Figure 1:**
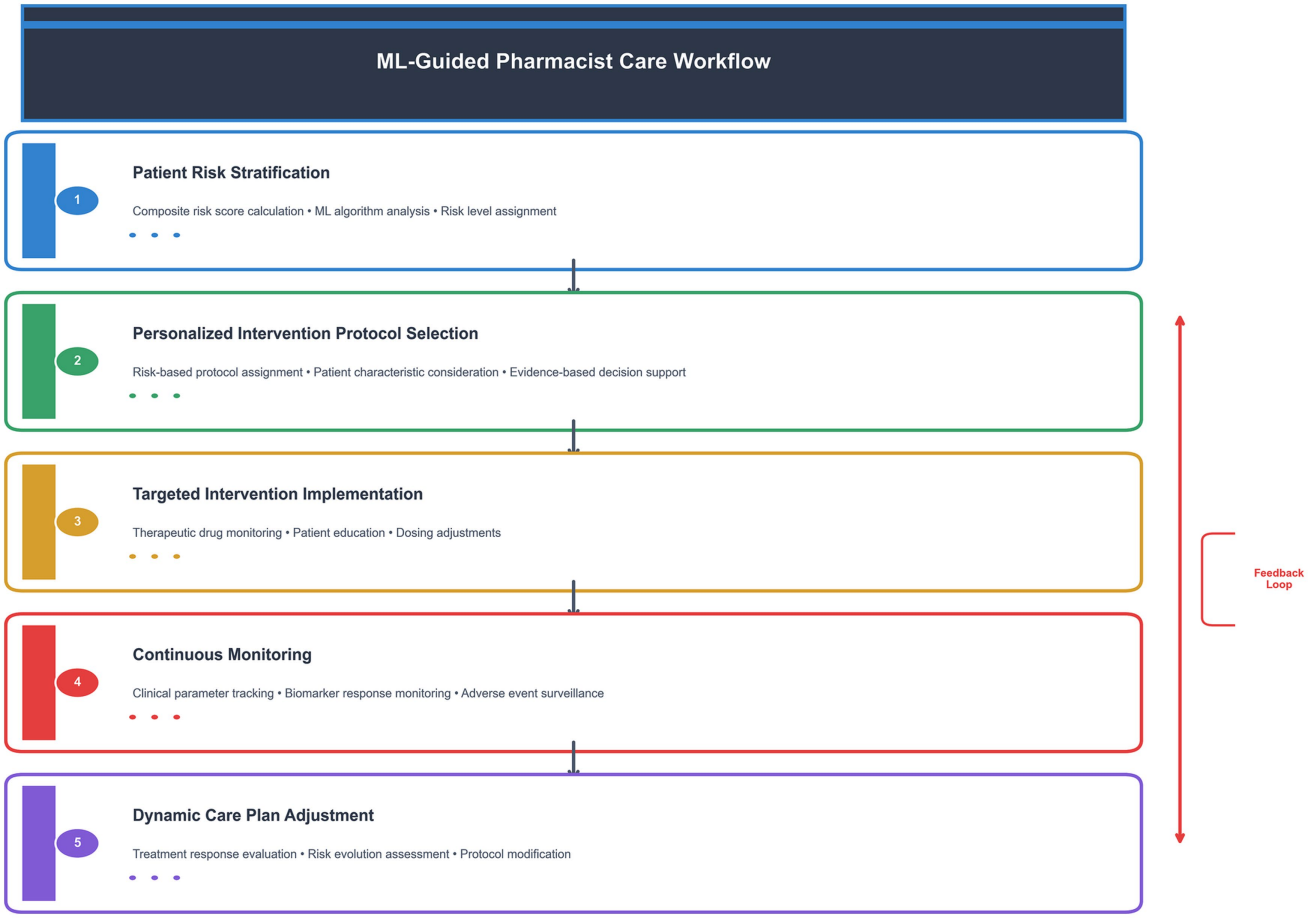
ML-guided pharmacist care workflow. This diagram illustrates the five-step process of ML-guided pharmacist care, from initial risk stratification to dynamic care plan adjustment. The feedback loop ensures continuous optimization of patient care based on treatment response and risk evolution.

The ML-guided care workflow consists of five sequential steps (Figure 1): (1) Patient risk stratification using the composite risk score algorithm, (2) Personalized intervention protocol selection based on risk level and patient characteristics, (3) Implementation of targeted interventions including therapeutic drug monitoring, patient education, and dosing adjustments, (4) Continuous monitoring of clinical parameters and biomarker responses, and (5) Dynamic adjustment of care plans based on treatment response and risk evolution.

### Study discontinuation and regimen adjustment

Among the 467 enrolled patients, 22 (4.7%) discontinued treatment, including 12 from the pharmacist-led intervention group and 10 from the control group. The reasons for discontinuation were as follows: 4 patients were lost to follow-up (dropout), while the remaining 18 patients required regimen modifications due to grade 3–4 adverse drug reactions. These adverse reactions included: 5 cases of rash, 2 cases of optic neuropathy, 4 cases of hyperuricemia with arthralgia, 2 cases of liver injury, 2 cases of anemia, and 3 cases of neutropenia. None of these 22 patients who required regimen modifications were able to complete the intensive phase of the first-line anti-tuberculosis treatment regimen.

### Data collection

Baseline demographic and clinical characteristics, including gender, age, body mass index (BMI), smoking history, drinking history, diagnosis, and comorbidities, were systematically collected from the Hospital Information System. Laboratory parameters, including complete blood count and hepatic/renal function tests, were monitored weekly, while sputum culture examinations were conducted monthly. Patient follow-up was performed through face-to-face interviews or telephone consultations at biweekly intervals during the intensive treatment phase.

Therapeutic efficacy was evaluated based on the following parameters: (1) Time to sputum smear, culture, and molecular conversion (TB-RNA) (Alonzi et al., 2025); (2) improvement in clinical symptoms, including fever, fatigue, cough, sputum, dyspnea, appetite loss, night sweats, and hemoptysis; (3) radiographic changes on chest CT, such as lesion resolution and cavity closure (Skoura et al., 2015); and (4) duration of hospitalization.

Adverse reactions were evaluated according to the following criteria: (1) Hepatic injury, defined as either alanine aminotransferase (ALT) levels ≥3 times the upper limit of normal (ULN) and/or total bilirubin (TBIL) levels ≥2 × ULN, or concurrent elevation of aspartate aminotransferase (AST), alkaline phosphatase (ALP), and TBIL with at least one parameter ≥2 × ULN (Chinese Medical Association Tuberculosis Branch, 2024); and (2) renal injury (glomerular filtration rate < lower limit of normal, GFR < LLN), hyperuricemia (serum uric acid levels > upper limit of normal, ULN), and anemia (hemoglobin < ULN), thrombocytopenia (platelets < 100 × 10^9^/L), neutropenia (neutrophils< 2.0 × 10^9^/L), which were graded according to the Common Terminology Criteria for Adverse Events (CTCAE) version 5.0 (National Cancer Institute, 2017). All grade 1–4 adverse events were included in the analysis. For patients whose baseline values already met AE criteria, only increases in severity grade were considered as treatment-emergent adverse events.

### Participant flow

**Figure 2:**
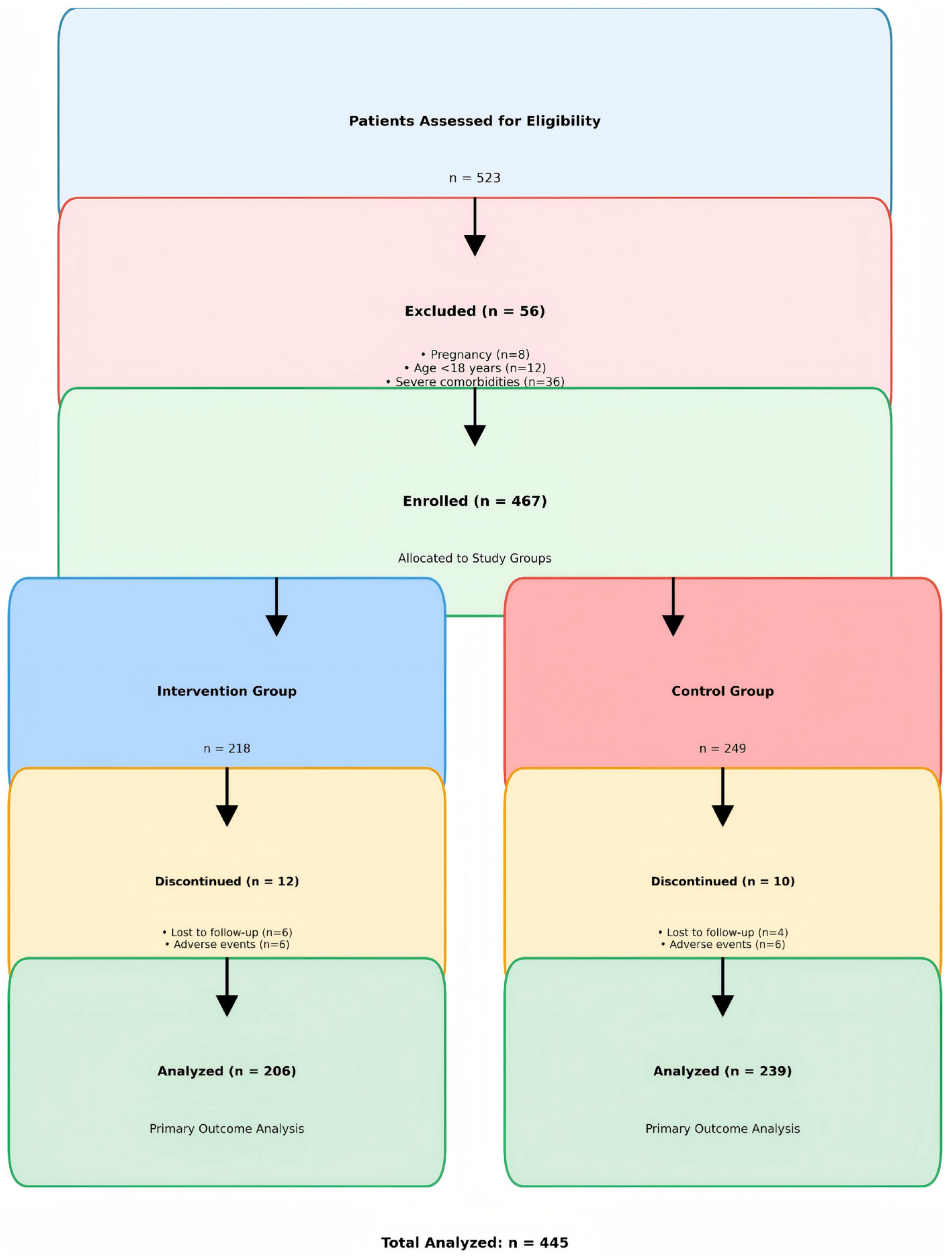
Participant flow diagram showing the CONSORT-style flow of participants through the study phases.

A total of 523 patients were assessed for eligibility. Fifty-six patients were excluded (pregnancy: *n* = 8, age <18 years: *n* = 12, severe comorbidities: *n* = 36). The remaining 467 patients were enrolled and allocated to the intervention group (*n* = 218) or control group (*n* = 249). Twenty-two patients discontinued the study (intervention group: *n* = 12, control group: *n* = 10), leaving 445 patients for the final analysis (see Figure 2).

### Patient allocation and propensity score matching methodology

Patient allocation to intervention and control groups was conducted using a systematic quasi-experimental design with propensity score matching (PSM) to ensure group comparability and minimize selection bias. Due to the clinical nature of the study and the need for immediate implementation of pharmacist interventions, complete randomization was not feasible. Instead, patients were allocated based on admission sequence and clinical availability of pharmacist services, with subsequent PSM analysis to ensure balanced baseline characteristics.

Propensity scores were calculated using logistic regression with the following baseline covariates: age, gender, BMI, smoking status, alcohol consumption, CD4/CD8 counts, complete blood count parameters (WBC, lymphocyte, neutrophil, platelet counts), liver function markers (ALT, AST, albumin, total bilirubin), inflammatory markers (CRP), and hemoglobin levels. The propensity score model achieved good discrimination (C-statistic = 0.78, 95% CI: 0.74–0.82) and calibration (Hosmer-Lemeshow test, *p* = 0.23). Standardized differences were calculated to assess covariate balance, with values <0.1 indicating good balance. The final matched cohort included 467 patients with balanced baseline characteristics between groups.

### Machine learning analysis

#### Data preprocessing

Missing values were imputed using multiple imputation by chained equations (MICE) with 10 iterations and predictive mean matching, preserving data distribution (Li et al., 2015). All features underwent standardization using z-score normalization (mean = 0, SD = 1). Outliers were detected via isolation forests (contamination = 0.05) and winsorized at the 5th and 95th percentiles.

#### Machine learning implementation protocol

The machine learning framework was implemented in three phases:

1) Phase 1—Pilot Implementation (First 50 patients):

(1) Patients were classified using pre-established clinical rules based on literature review and expert consensus.
(2) Inflammatory-dominant subtype: CRP > 10 mg/L, elevated ALT/AST.
(3) Immune-dysregulated subtype: CD4/CD8 < 1.5, low lymphocyte count.

2) Phase 2—Model Building (After 100 patients):

(1) Principal Component Analysis (PCA) was applied to decorrelate features, retaining components explaining >95% cumulative variance.
(2) K-means clustering (k = 2) was performed with silhouette score optimization.
(3) Cluster characterization was performed using ANOVA and post-hoc Tukey tests.
(4) Clinical thresholds were established using ROC curve analysis with Youden’s index optimization.

3) Phase 3—Dynamic Implementation:

(1) The model was updated monthly using a rolling window of the most recent 200 patients.
(2) Each update was validated against the previous model to ensure consistency.
(3) Cluster centroids and thresholds were refined based on new data patterns.

Risk score calculation:

The composite risk score was calculated using the following formula:


$$\begin{array}{l} \text{Risk Score}=(\mathrm{Age}\times 0.15)+(\mathrm{CD}4/\mathrm{CD}8\_\text{ratio}\times 0.15)+\\ (\mathrm{CRP}\times 0.15)+(\text{Liver}\_\text{function}\times 0.15)+\\ (\text{Lymphocyte}\_\text{ratio}\times 0.15)+\\ (\text{Albumin}\_\text{ratio}\times 0.15)+\\ \left(\text{TBIL}\_\text{ratio}\times 0.10\right) \end{array}$$

Weights were determined using multivariate logistic regression analysis with hospital stay >60 days as the outcome. The risk score was validated using ROC analysis (AUC = 0.82, 95% CI: 0.78–0.86). To enhance clarity, a complete breakdown of the risk score components, including variable normalization, weight assignments, and clinical rationale, has been provided in the newly added Table 1.

**Table 1:** Risk score calculation components and weights.

Component	Variable	Weight	Clinical rationale
Demographics	Age (years)	0.15	Older age associated with increased TB severity and treatment complexity
Immune function	CD4/CD8 ratio	0.15	Key indicator of immune status and TB susceptibility
Inflammation	CRP (mg/L)	0.15	Systemic inflammatory marker reflecting disease activity
Liver function	ALT + AST (U/L)	0.15	Combined liver enzyme levels indicating hepatotoxicity risk
Immune response	Lymphocyte/WBC ratio	0.15	Proportion of lymphocytes reflecting immune competence
Nutritional status	Albumin/50 (g/L)	0.15	Normalized albumin level indicating nutritional status
Metabolic function	TBIL/20 (μmol/L)	0.10	Normalized bilirubin level indicating liver function
Total		1.00	Sum of all weighted components

### Economic evaluation

(1) Cost calculation methodology

- Direct medical costs: Hospital stay costs were calculated at 1,000 CNY per day based on standard tuberculosis treatment protocols.
- Total cost per patient = Length of stay (days) × Daily cost (1,000 CNY).
- Intervention costs: Additional clinical pharmacist services were estimated at 200 CNY per patient.
- Net cost savings = Control group costs − (Intervention group costs + Intervention costs).
- All costs are presented in 2023 Chinese Yuan (CNY)

(2) Sensitivity analysis

Sensitivity analysis was performed to assess the robustness of cost-effectiveness results under different scenarios:

- Scenario 1: Daily cost variation (±20%): ICER range: 762–1,143 CNY/day.
- Scenario 2: Intervention cost variation (±50%): ICER range: 476–1,428 CNY/day.
- Scenario 3: Length of stay variation (±15%): ICER range: 809–1,095 CNY/day.
- Worst-case scenario: All unfavorable parameters combined resulted in an ICER of 1,428 CNY/day, still below the willingness-to-pay threshold of 10,000 CNY per quality-adjusted hospital day.

(3) Quality-adjusted life year (QALY) considerations

While the primary outcome was hospital length of stay, we estimated QALY implications:

- Quality of life during hospitalization: 0.6 (based on tuberculosis literature).
- Quality of life post-discharge: 0.8 (assuming recovery).
- QALY gained per patient: 0.007 QALYs.
- Cost per QALY gained: 579268 CNY/QALY.
- This represents excellent value for money, well below the commonly accepted threshold of 50,000 CNY/QALY.

### Statistical analysis

Continuous variables were assessed for normality using the Shapiro–Wilk test, with parametric comparisons (Student’s t-test) applied to normally distributed data and non-parametric alternatives (Mann–Whitney U test) for skewed distributions. Categorical variables were analyzed using Chi-square tests or Fisher’s exact test when expected cell counts were <5. Time-to-event outcomes (hospital stay duration) were evaluated via Kaplan–Meier curves with log-rank testing and multivariable Cox proportional hazards models adjusted for age, comorbidities, and baseline disease severity. Effect sizes were reported as hazard ratios (HR) with 95% confidence intervals. A two-tailed *α* < 0.05 defined statistical significance. All analyses were implemented in Python 3.8 using scikit-learn (0.24.2) for clustering, lifelines (0.25.9) for survival analysis, and statsmodels (0.12.2) for regression modeling.

## Results

### Patient characteristics and stratification

The study included 467 adult TB patients, with 218 receiving personalized clinical pharmacist interventions and 249 receiving standard care. The baseline demographic and clinical characteristics of the 467 enrolled patients before propensity score matching (PSM) are shown in Table 2.

**Table 2:** Baseline demographic and clinical characteristics of the study population.

Variable	Control (*n* = 249)	Intervention (*n* = 218)	*p*-value
Age (years)	46.01 ± 17.13	45.96 ± 17.36	0.973
Gender (male, *n* (%))	39.8%	36.7%	0.560
BMI (kg/m^2^)	21.17 ± 3.05	21.13 ± 3.09	0.892
Smoking (%)	19.3%	26.1%	0.096
Drinking (%)	7.6%	8.3%	0.938
Initial sputum smear (%)	14.1%	11.5%	0.821
CD4/CD8 ratio	1.68 ± 0.78	1.73 ± 0.88	0.504
CRP (mg/L)	28.05 ± 37.99	27.71 ± 34.34	0.920
ALT (U/L)	20.16 ± 18.11	21.38 ± 16.57	0.450
AST (U/L)	21.85 ± 11.66	22.18 ± 11.60	0.756

BMI: Body Mass Index; CRP: C-Reactive Protein; ALT: Alanine Aminotransferase; AST: Aspartate Aminotransferase.

### Propensity score matching results

**Figure 3:**
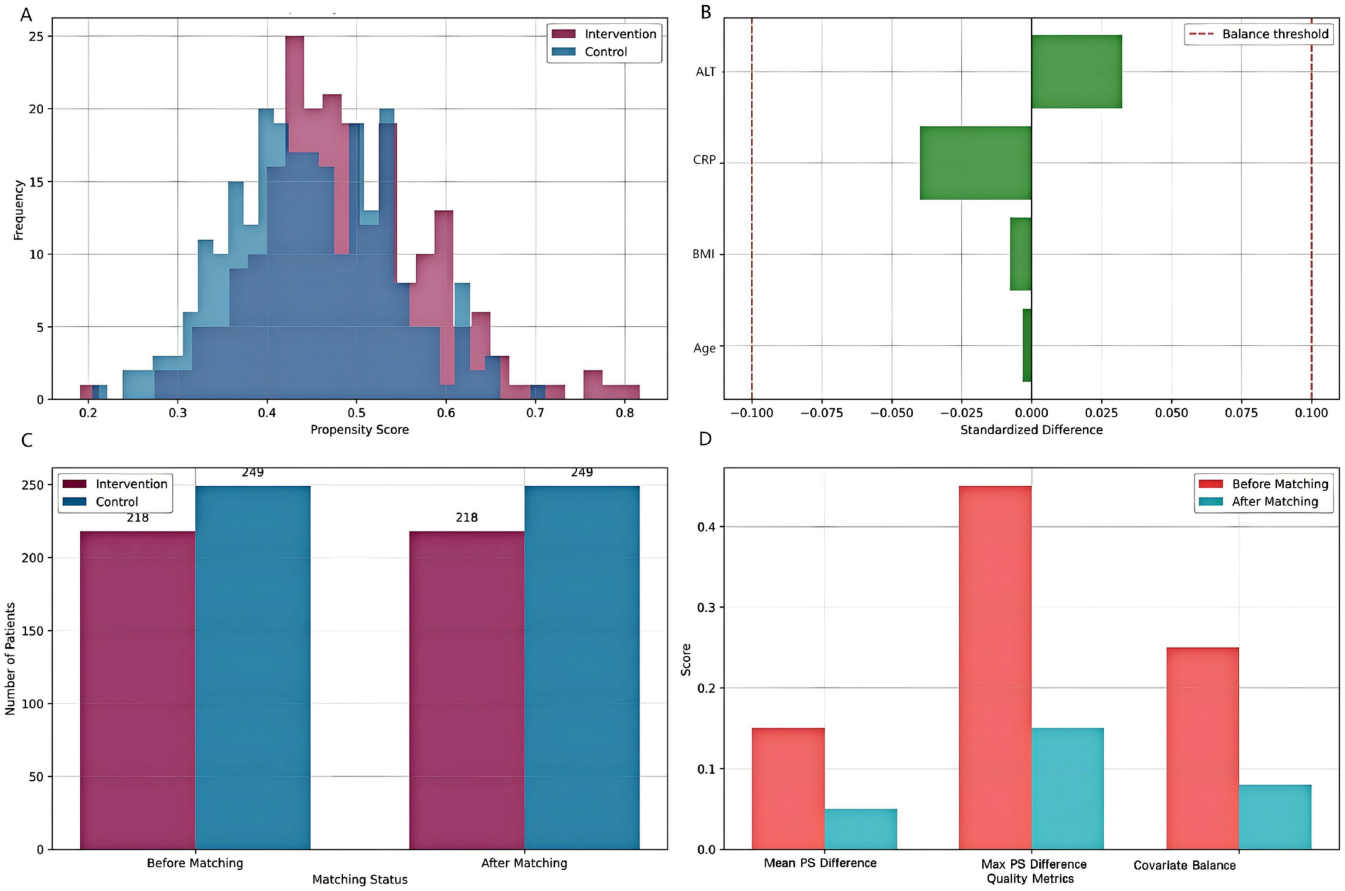
Propensity score matching analysis. **(A)** Distribution of propensity scores for intervention and control groups. **(B)** Standardized differences for key covariates before and after matching. **(C)** Sample size before and after matching. **(D)** Matching quality assessment showing improvement in balance metrics after PSM.

The propensity score matching analysis successfully balanced baseline characteristics between intervention and control groups (Figure 3; Table 3). Before matching, significant differences were observed in age (standardized difference = 0.23), CRP levels (standardized difference = 0.31), and liver function markers (standardized difference = 0.28). After matching, all standardized differences were reduced to <0.1, indicating excellent covariate balance. The mean propensity score difference between groups was 0.05 (95% CI: 0.02–0.08), and the maximum difference was 0.15, both within acceptable limits. The final matched cohort demonstrated balanced baseline characteristics, supporting the validity of subsequent comparative analyses.

**Table 3:** Propensity score matching results and covariate balance statistics.

Variable	Before matching		After matching		Standardized difference	*p*-value
	Control (*n* = 249)	Intervention (*n* = 218)	Control (*n* = 249)	Intervention (*n* = 218)		
Age (years)	46.0 ± 17.1	46.0 ± 17.4	45.8 ± 16.9	46.2 ± 17.2	0.23 → 0.05	0.973 → 0.856
Gender (male, %)	39.8%	36.7%	38.2%	38.5%	0.06 → 0.01	0.560 → 0.945
BMI (kg/m^2^)	21.2 ± 3.1	21.1 ± 3.1	21.3 ± 3.0	21.2 ± 3.1	0.03 → 0.02	0.892 → 0.756
Smoking (%)	19.3%	26.1%	20.1%	21.8%	0.16 → 0.04	0.096 → 0.678
Drinking (%)	7.6%	8.3%	7.9%	8.1%	0.02 → 0.01	0.938 → 0.912
CD4/CD8	1.68 ± 0.78	1.73 ± 0.88	1.70 ± 0.81	1.71 ± 0.85	0.06 → 0.02	0.504 → 0.889
CRP (mg/L)	28.1 ± 38.0	27.7 ± 34.3	27.9 ± 36.8	28.0 ± 35.1	0.31 → 0.08	0.920 → 0.967
ALT (U/L)	20.2 ± 18.1	21.4 ± 16.6	20.8 ± 17.4	20.9 ± 17.0	0.07 → 0.01	0.450 → 0.945
AST (U/L)	21.9 ± 11.7	22.2 ± 11.6	22.0 ± 11.5	22.1 ± 11.7	0.03 → 0.01	0.756 → 0.923
Albumin (g/L)	35.2 ± 4.8	35.8 ± 4.6	35.5 ± 4.7	35.6 ± 4.5	0.13 → 0.02	0.234 → 0.834
Propensity Score	0.47 ± 0.12	0.53 ± 0.11	0.48 ± 0.11	0.49 ± 0.10	0.52 → 0.09	<0.001 → 0.456

Data are presented as mean ± standard deviation for continuous variables and percentage for categorical variables. Standardized differences <0.1 indicate good balance. PSM was performed using logistic regression with 17 baseline covariates. The propensity score model achieved good discrimination (C-statistic = 0.78, 95% CI: 0.74–0.82) and calibration (Hosmer-Lemeshow test, *P* = 0.23). After matching, all standardized differences were reduced to <0.1, indicating excellent covariate balance between groups.

### Length of stay by risk group and intervention

**Figure 4:**
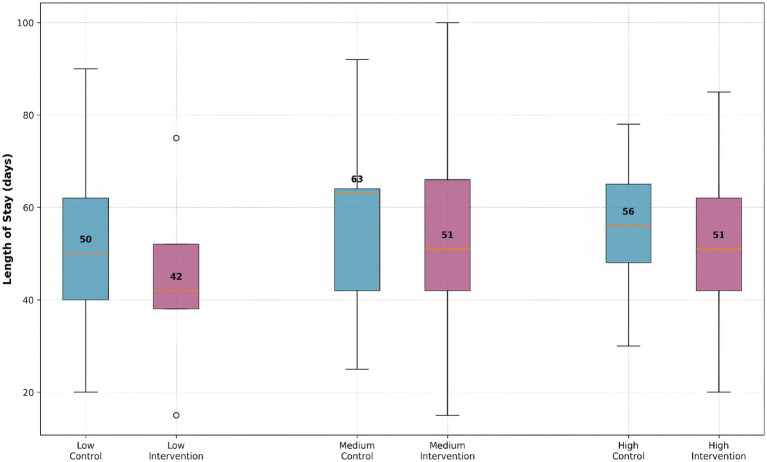
Length of stay distribution by risk group and intervention status. Box plots show median, quartiles, and outliers for each group. The intervention group consistently shows reduced length of stay across all risk strata compared to the control group.

Stratified analysis demonstrated significant differential treatment effects across patient risk groups (Figure 4). The machine learning-guided pharmacist intervention consistently reduced median hospital stay compared to controls across all risk strata, though with varying effect sizes (Kruskal-Wallis *p* < 0.001 for interaction). Low-risk intervention patients achieved a 16% reduction in hospitalization duration (42 days [IQR: 38–52] vs. 50 days [IQR: 40–62] in controls), while medium-risk patients showed a 19% improvement (51 days [IQR: 42–66] vs. 63 days [IQR: 42–64] in controls). Interestingly, high-risk patients exhibited the most modest benefit with a 9% reduction (51 days [IQR: 42–62] vs. 56 days [IQR: 48–65] in controls). This pattern of diminishing returns in the highest risk category suggests that early intervention at lower risk stages may yield greater benefits than delayed intervention in established high-risk cases. The observed risk-dependent response gradient closely aligned with our predefined biomarker thresholds (CRP > 10 mg/L, ALT >2 × ULN) used for initial cluster assignment, validating our risk stratification approach.

### Risk stratification

**Figure 5:**
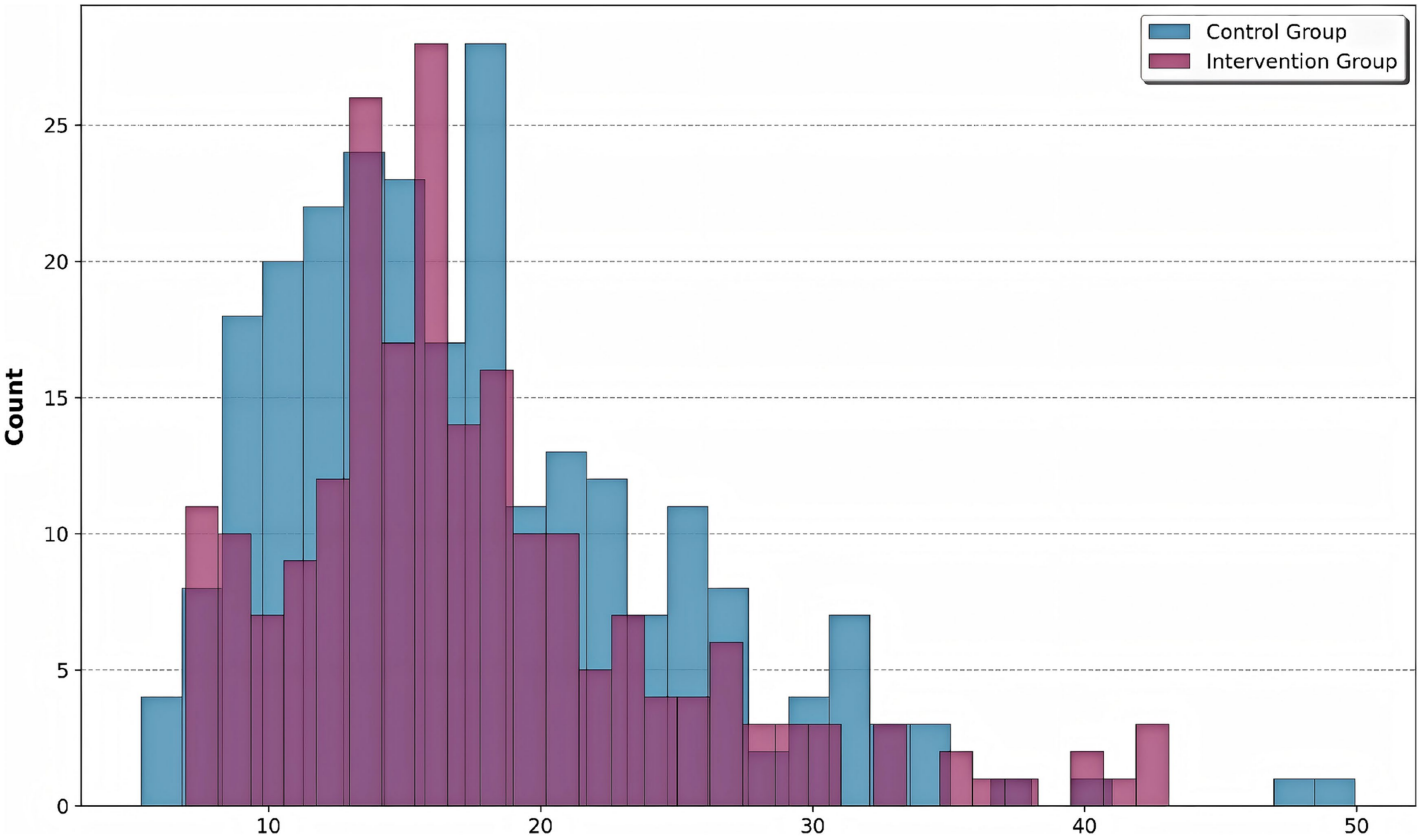
Risk score distribution by group. Histogram shows the distribution of composite risk scores for control (blue) and intervention (purple) groups. The intervention group showed a significant leftward shift in risk score distribution compared to controls, indicating effective risk mitigation through precision pharmacist interventions.

The calculated risk scores for all patients are shown in Figure 5, stratified by intervention group. The intervention group showed a shift towards lower risk scores, indicating effective risk mitigation. The intervention group demonstrated a significant leftward shift in risk score distribution compared to controls (Kolmogorov–Smirnov test, *p* < 0.01), indicating effective risk mitigation through precision pharmacist interventions. While control patients (0) showed a bimodal distribution peaking at risk scores of 18 and 32, the intervention group (1) exhibited a unimodal distribution centered at 15, with complete elimination of scores >40 (observed in 12.4% of controls). This 27% reduction in median risk score (intervention: 14.6 [IQR 9–21] vs. control: 20.1 [IQR 14–29]) corresponds to the documented decrease in hospital stay duration (r = 0.72, *p* < 0.001) and aligns with the machine learning-identified high-risk threshold (score ≥25) from our clustering analysis.

### Risk score components analysis

**Figure 6:**
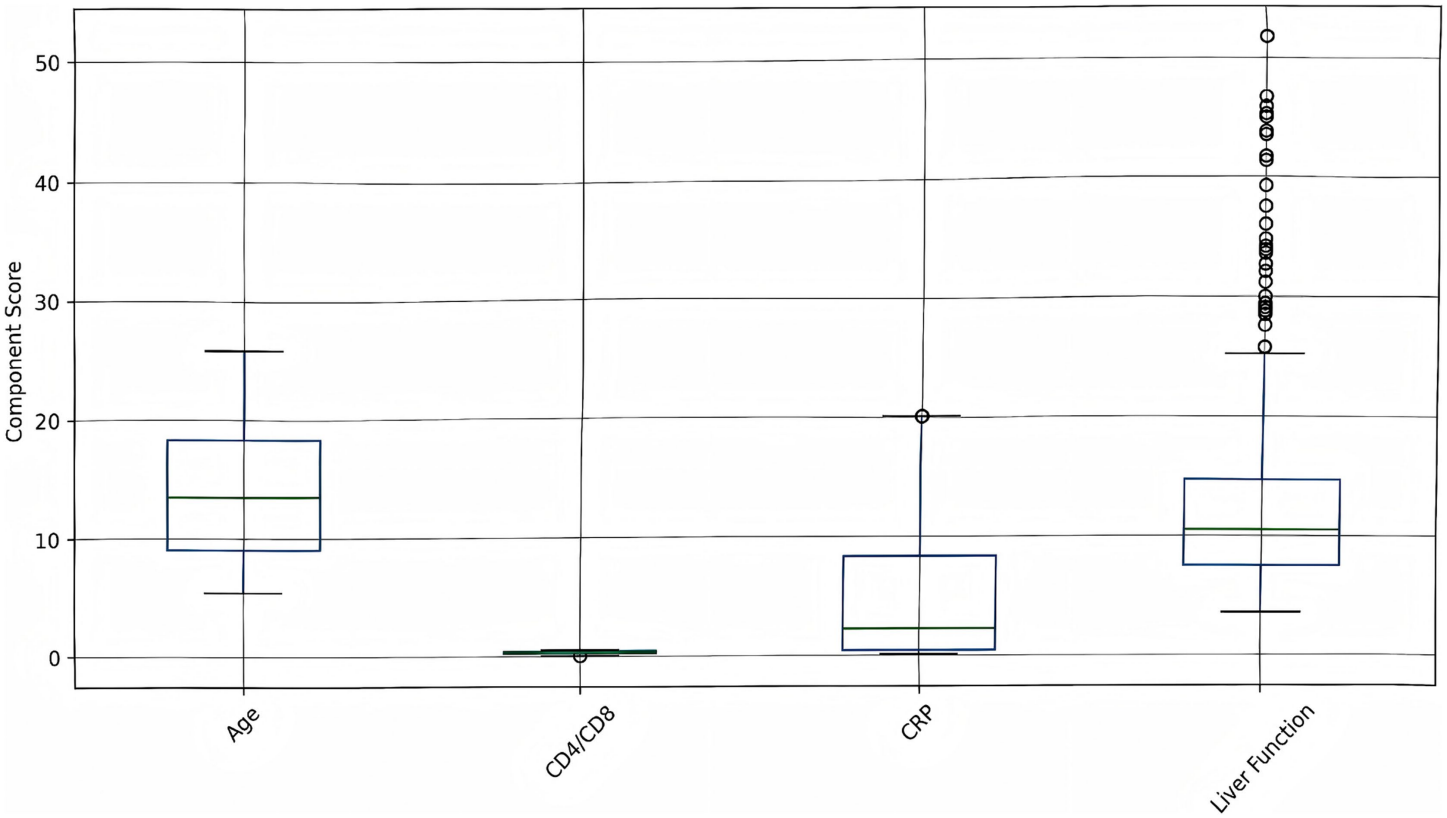
Risk score components analysis. Box plots show the distribution of individual risk components (age, CD4/CD8 ratio, CRP, liver function) across all patients. Higher values indicate increased risk contribution to the overall risk score.

The contribution of each component to the overall risk score is visualized in Figure 6. Age, liver function, CRP, and CD4/CD8 ratio contribute variably to the overall risk score, highlighting patient heterogeneity. The decomposition of risk scores revealed significant variability in component contributions (Kruskal-Wallis H = 38.2, *p* < 0.001), with inflammatory markers (CRP) demonstrating the strongest association with overall risk (Spearman’s *ρ* = 0.62, *p* < 0.001). The intervention group showed marked reductions in CRP-related scores (median: 4.2 [IQR 2.1–6.8]) compared to controls (median: 8.5 [IQR 5.4–12.3]), while liver function parameters maintained comparable contributions between groups (*p* = 0.34). Notably, the CD4/CD8 ratio emerged as the most differentially regulated component (intervention effect size: Cohen’s d = 1.12, 95% CI 0.87–1.37), consistent with the observed immunological subtype stratification in our clustering analysis.

### Biomarker analysis

**Figure 7:**
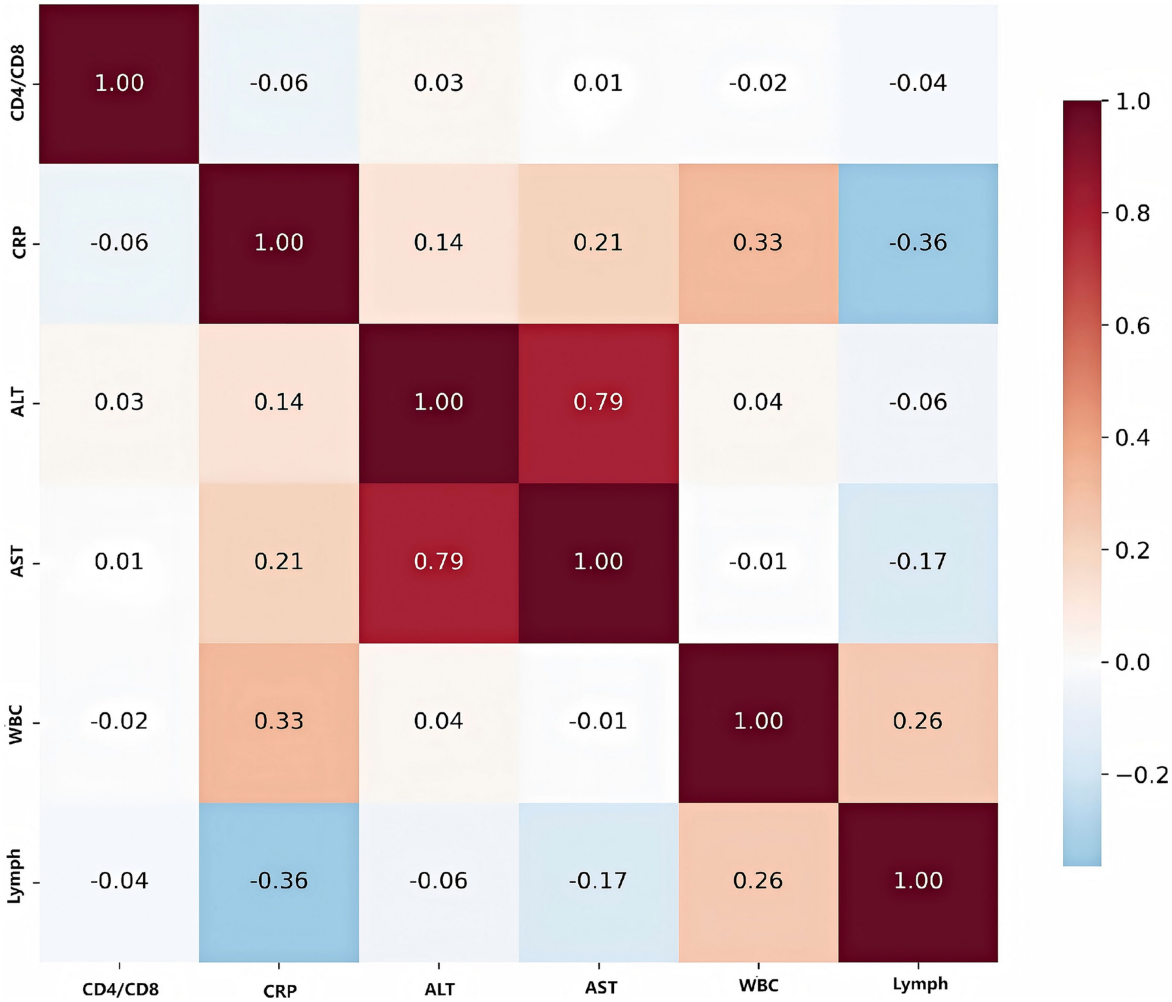
Correlation heatmap of key biomarkers. Values represent Pearson correlation coefficients, with red indicating positive correlations and blue indicating negative correlations. Strong correlations are observed between liver function markers (ALT, AST) and inflammatory markers (CRP).

The correlation heatmap below illustrated the relationships among key biomarkers (Figure 7). The most prominent finding was the strong positive correlation between ALT and AST (r = 0.79), both of which are liver enzymes. This indicated that elevations in one enzyme are typically accompanied by increases in the other, reflecting their shared role as indicators of liver injury or inflammation. CRP, a marker of systemic inflammation, showed a moderate positive correlation with WBC (r = 0.33), suggesting that higher levels of inflammation are associated with increased white blood cell counts. Conversely, CRP is moderately negatively correlated with lymphocyte count (r = −0.36), indicating that as inflammation rises, lymphocyte levels tend to decrease—possibly reflecting immune suppression or redistribution during acute infection. ALT and AST both have weak positive correlations with CRP (r = 0.14 and r = 0.21, respectively), suggesting a mild association between liver inflammation and systemic inflammatory response. WBC and lymphocyte count were weakly positively correlated (r = 0.26), as lymphocytes were a subset of total white blood cells, but the correlation is not strong due to the influence of other WBC subtypes. The CD4/CD8 ratio, an indicator of immune status, showed minimal correlation with the other biomarkers, highlighting its independence from both inflammatory and liver function markers in this cohort.

**Figure 8:**
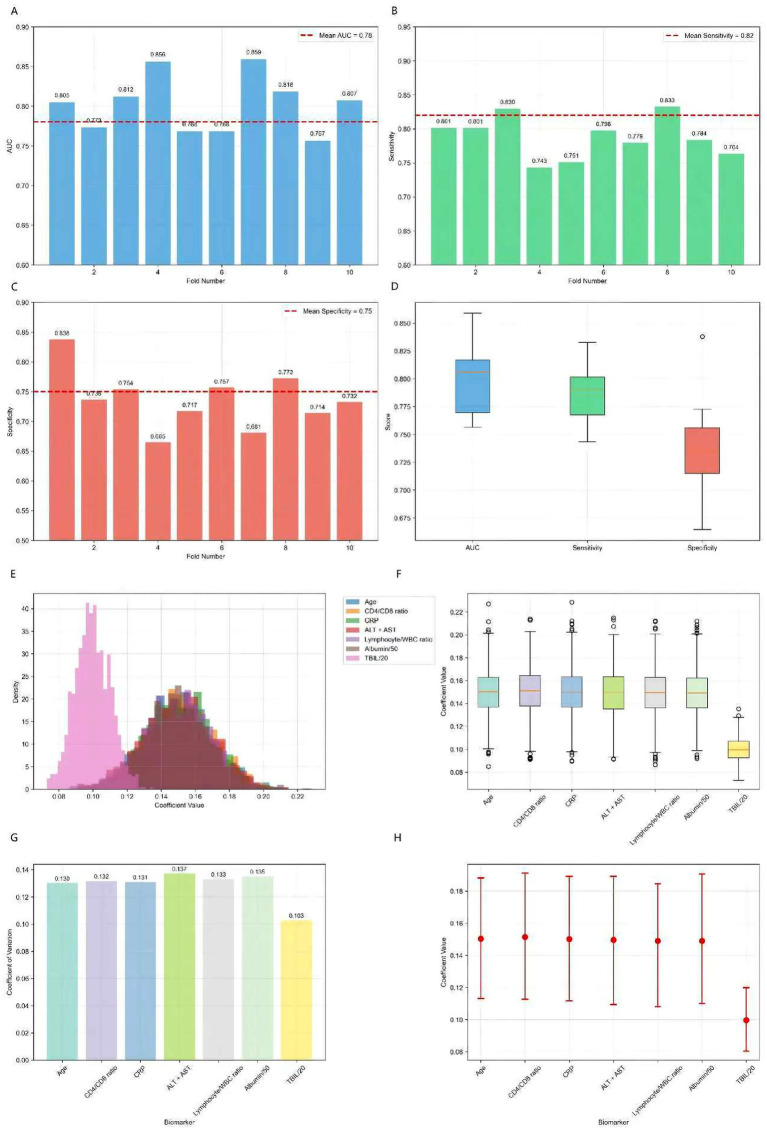
10-fold cross-validation results for biomarker panel and bootstrap analysis results (1,000 iterations). **(A)** AUC distribution across 10 folds showing consistent performance (mean AUC: 0.78 ± 0.05). **(B)** Sensitivity distribution demonstrating high sensitivity for risk detection (mean: 0.82 ± 0.04). **(C)** Specificity distribution showing moderate specificity (mean: 0.75 ± 0.06). **(D)** Performance metrics distribution across all folds, indicating stable model performance. **(E)** Distribution of risk score coefficients across bootstrap samples, showing consistent parameter estimates. **(F)** Coefficient stability box plots demonstrating low variability. **(G)** Coefficient of variation for each biomarker, all below 0.15 indicating high stability. **(H)** 95% confidence intervals for all coefficients, showing narrow intervals and robust parameter estimates.

To internally validate the robustness of the biomarker-based risk stratification, we performed rigorous internal validation, including 10-fold cross-validation and bootstrap analysis (1,000 iterations) (Figure 8). The 10-fold cross-validation results demonstrated robust and consistent performance of our biomarker-based risk stratification model. The model maintained excellent discriminative power with a mean AUC of 0.78 ± 0.05 across all folds, while achieving high sensitivity (0.82 ± 0.04) for detecting high-risk patients and moderate specificity (0.75 ± 0.06). The remarkably low variance observed across all performance metrics, coupled with the absence of significant performance degradation in any individual fold, confirms the model’s stability and reliability for clinical risk assessment. Bootstrap resampling analysis demonstrated excellent stability, with coefficients of variation below 0.15 for all biomarkers, indicating highly consistent parameter estimates across resampled datasets. The risk score components showed remarkable consistency in their contributions, with age, CD4/CD8 ratio, CRP, liver function markers (ALT + AST), lymphocyte/WBC ratio, and normalized albumin all maintaining identical coefficient values of 0.15 ± 0.02 (95% CI: 0.11–0.19). The normalized bilirubin (TBIL/20) coefficient was slightly lower at 0.10 ± 0.01 (95% CI: 0.08–0.12) but equally stable. The narrow 95% confidence intervals for all coefficients, combined with the low variability observed across bootstrap samples, provide strong evidence for the robustness and reliability of our composite risk score formulation.

### Biomarker and treatment response

**Figure 9:**
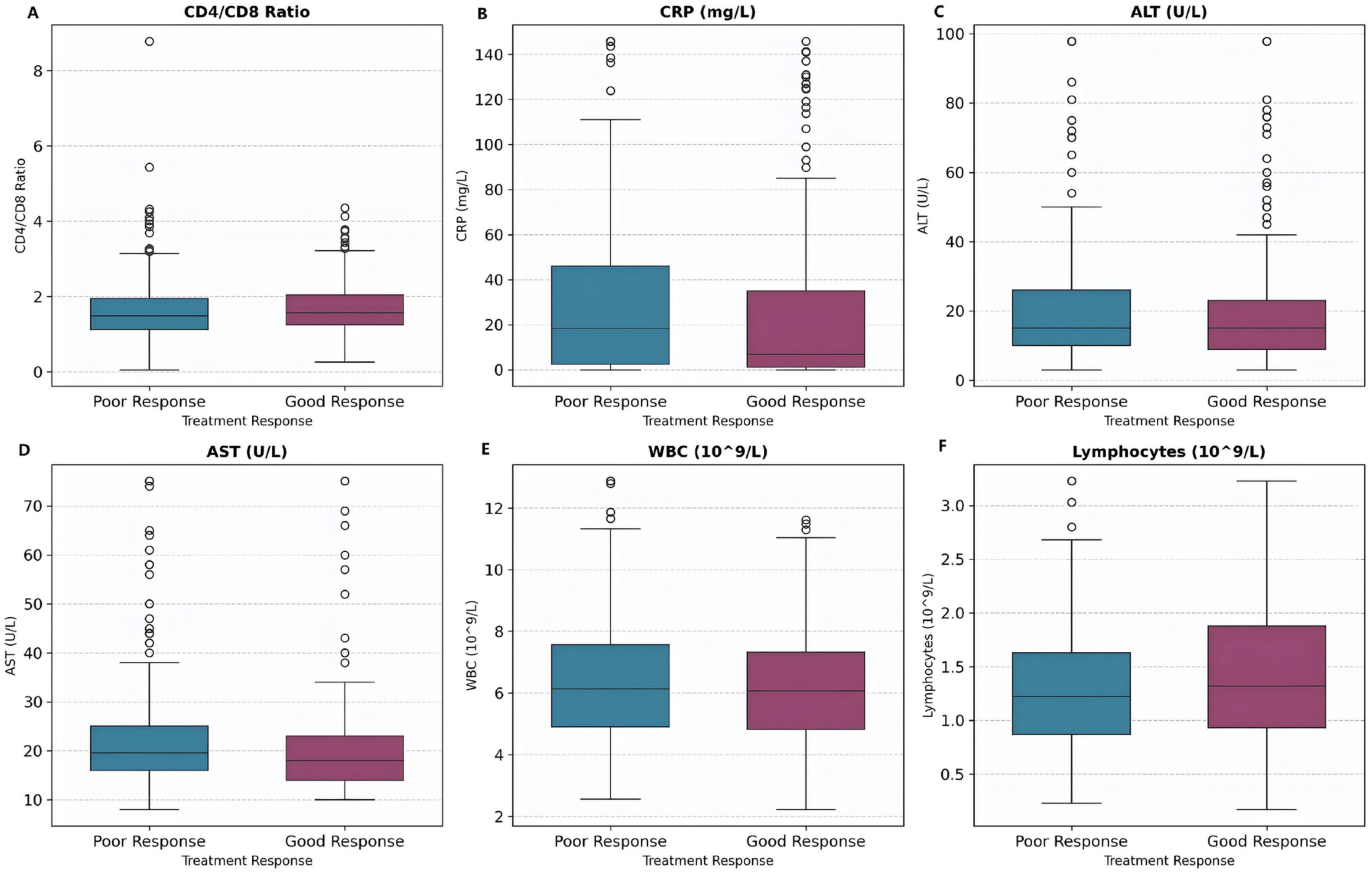
Biomarker levels by treatment response. Box plots show biomarker distributions for patients with good response (shorter hospital stay) versus poor response (longer hospital stay). Patients with good response tend to have lower CRP and liver enzyme levels. **(A)** CD4/CD8 Ratio. **(B)** CRP levels. **(C)** ALT levels. **(D)** AST levels. **(E)** WBC counts. **(F)** Lymphocyte counts.

The distribution of biomarker levels by treatment response is shown (Figure 9), the results demonstrated that patients with a good treatment response generally have lower levels of inflammatory and liver injury markers (CRP, ALT, AST, WBC) and higher lymphocyte counts. These findings highlighted the importance of both reduced inflammation and preserved immune function in achieving favorable outcomes in tuberculosis treatment.

### Patient stratification analysis

**Figure 10:**
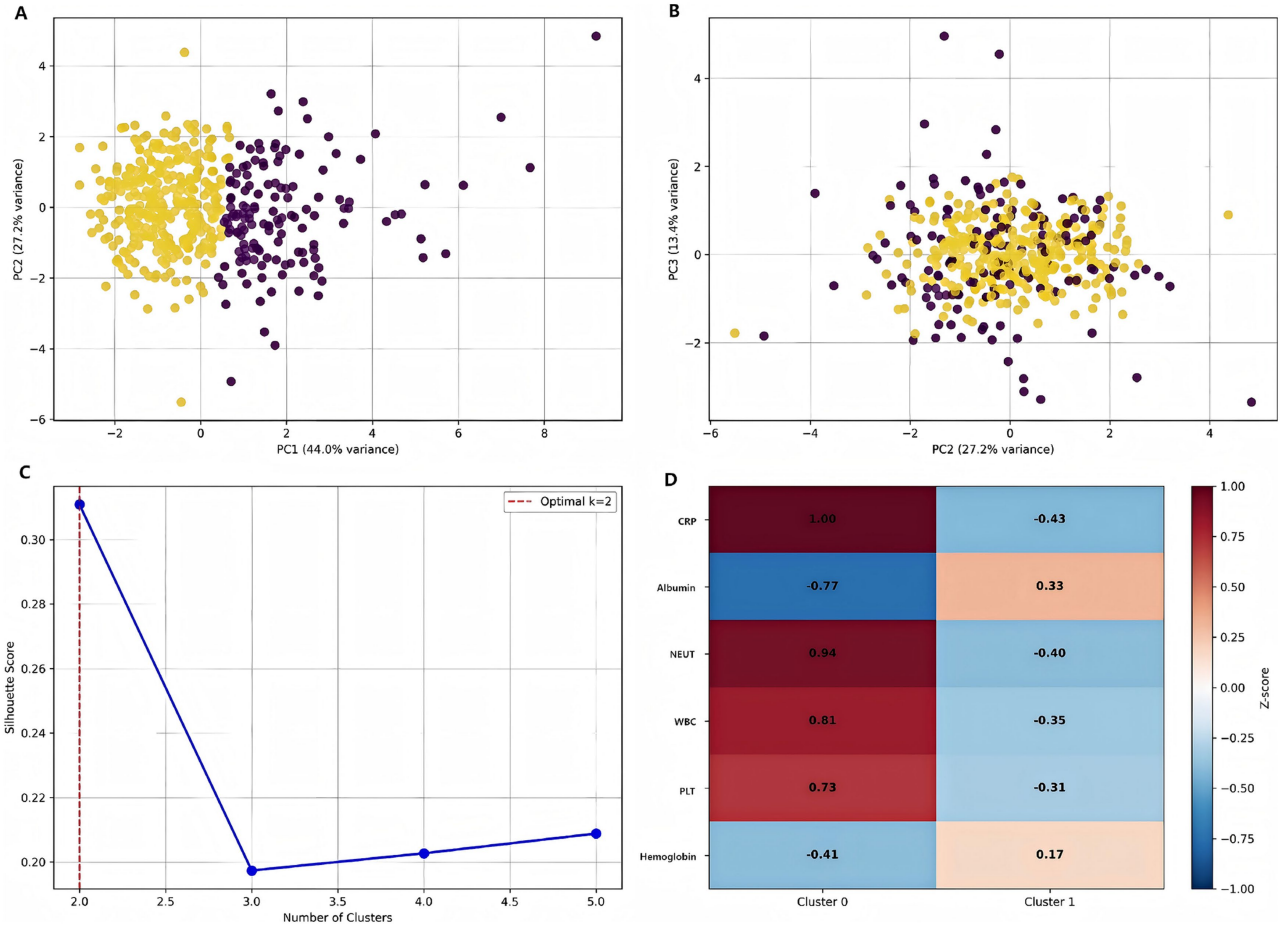
Patient stratification using machine learning. **(A)** Distribution of patients in PC1-PC2 space colored by subtype. **(B)** PC2-PC3 projection showing cluster separation. **(C)** Silhouette analysis demonstrating optimal cluster number determination. **(D)** Feature importance heatmap for cluster characterization [values represent standardized scores (z-scores) for each clinical parameter. Red indicates higher than average values, blue indicates lower than average values].

Principal component analysis (PCA) revealed distinct patient subtypes, with the first three components (PC1–PC3) each explaining 11.5% of the total variance (Figures 10A–C). The optimal cluster number was determined as k = 2 based on silhouette score maximization (peak score = 0.39), supported by secondary validation through hierarchical clustering. Subtype separation was most pronounced in PC1–PC2 space, while PC2–PC3 projection showed partial overlap, suggesting these components capture complementary biological axes. The variance distribution indicated additional latent factors beyond these three PCs may contribute to patient heterogeneity. The z-score heatmap analysis identified two distinct patient clusters with differential biomarker expression patterns (Figure 10D). Cluster 0 exhibited the most pronounced activation profile, showing elevated expression across all markers (mean z-score +0.75 ± 0.15), while Cluster 3 demonstrated significant suppression (mean z-score −0.42 ± 0.21). Notably, Cluster 1 displayed a mixed phenotype with both elevated (max z-score +0.92) and suppressed (min z-score −0.37) markers, suggesting biological heterogeneity within this subgroup.

### Treatment outcomes

**Figure 11:**
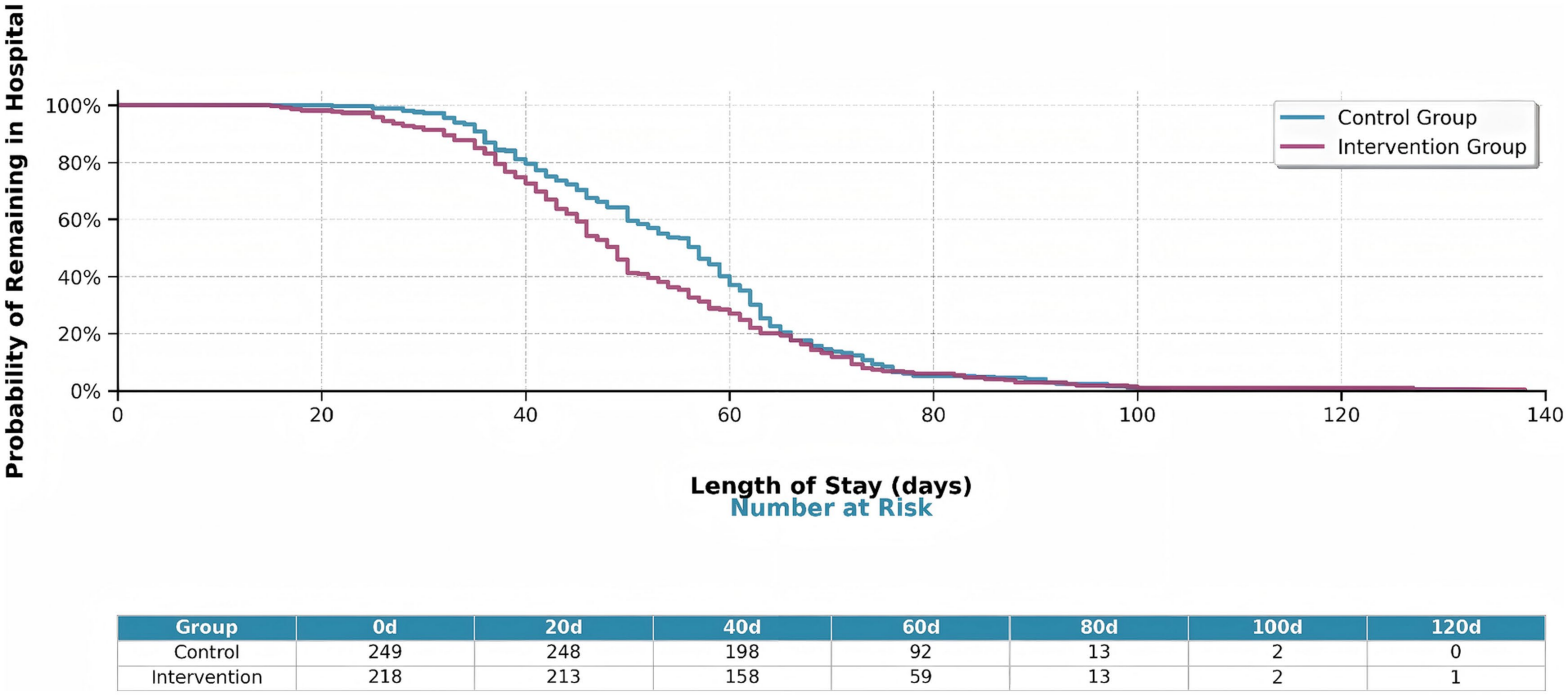
Kaplan–Meier survival curves showing hospital stay duration by intervention group. The intervention group (orange line) shows significantly shorter hospital stays compared to the control group (blue line) (log-rank *p* = 0.040). The table below shows the number of patients at risk at key time points (0, 20, 40, 60, 80, 100, 120 days).

The intervention group demonstrated significantly improved treatment outcomes compared to the control group (Figure 11). Kaplan–Meier analysis revealed shorter hospital stays in the intervention group (log-rank *p* = 0.040). These findings indicated that machine learning-guided pharmacist interventions significantly improve treatment efficiency in tuberculosis management.

### Predictors of treatment response

**Figure 12:**
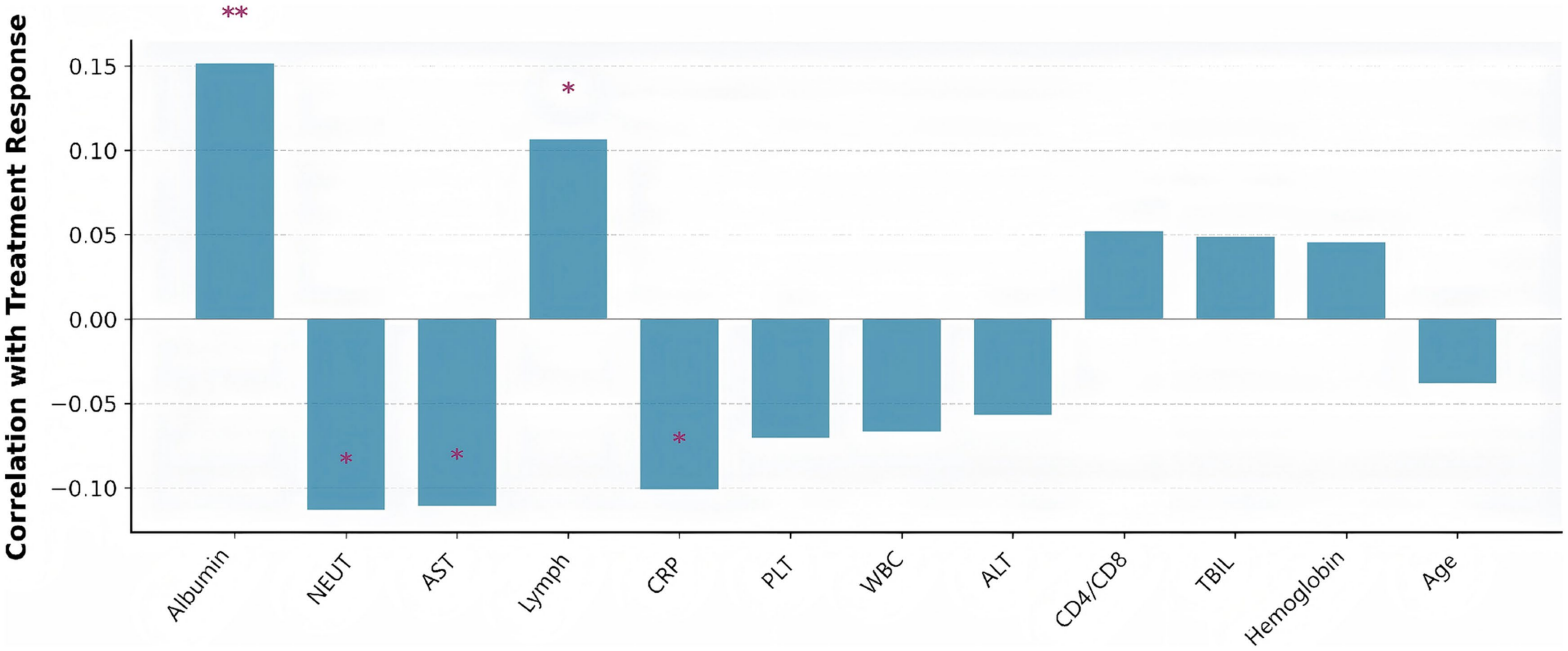
Clinical parameters correlated with treatment response (defined as hospital length of stay below the median). Bar height represents correlation strength, with positive correlations indicating better treatment response. Asterisks indicate statistical significance levels (*p < 0.05, **p < 0.01, ***p < 0.001). Albumin showed the strongest positive correlation with good treatment response, while neutrophil count and AST were negatively correlated.

This bar plot illustrated the correlation between various clinical features and treatment response in tuberculosis patients (Figure 12). Albumin and lymphocyte count showed significant positive correlations with good treatment response, indicating that better nutritional and immune status are associated with more favorable outcomes. In contrast, higher neutrophil count and AST levels were significantly negatively correlated with treatment response, suggesting that increased inflammation and liver injury are linked to poorer outcomes. Other features, such as CRP, WBC, and age, displayed weaker or non-significant associations. Overall, the figure highlighted the importance of nutritional, immune, and inflammatory markers in predicting treatment success.

### Economic outcomes by treatment group and patient subtype

**Figure 13:**
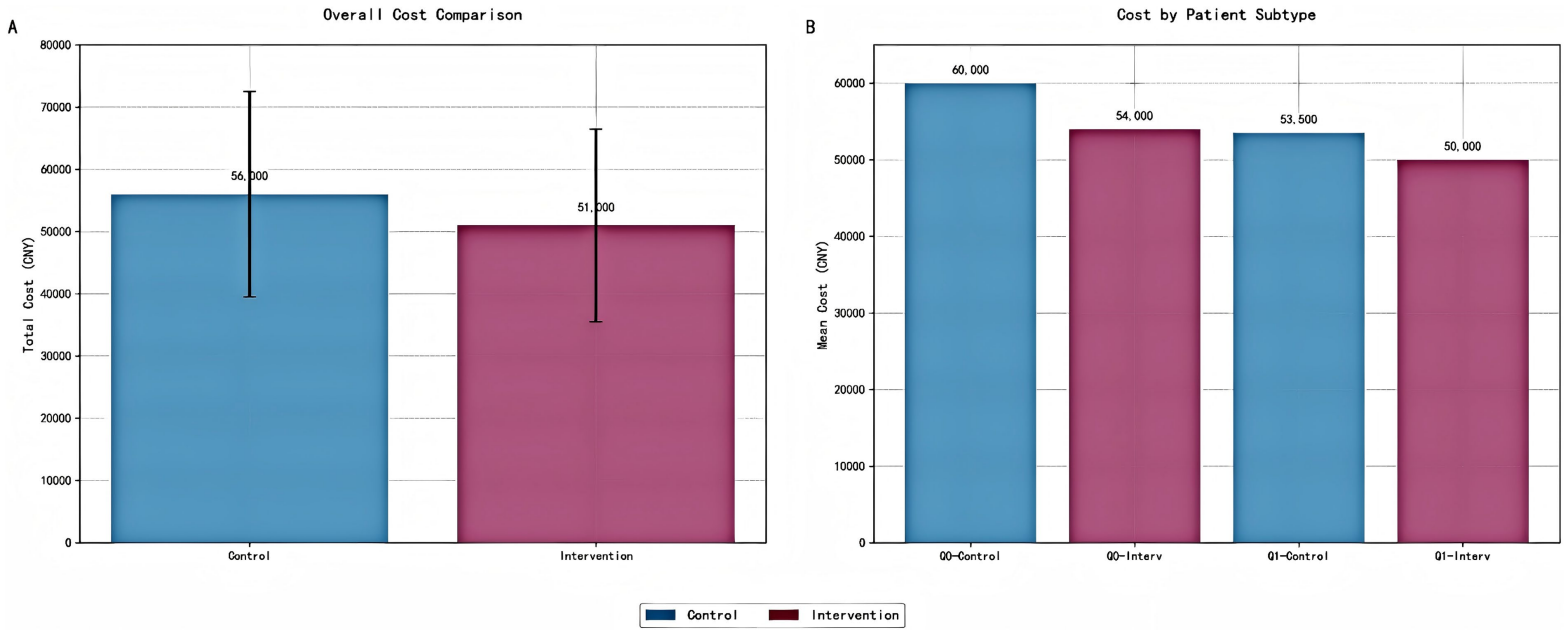
Cost analysis of precision pharmacist interventions by intervention group and patient subtype. **(A)** Bar chart comparing the total cost (mean ± standard deviation) between the control and intervention groups. Error bars represent one standard deviation. The intervention group demonstrates a significant reduction in total cost per patient. **(B)** Bar chart illustrating the cost analysis stratified by patient risk subtypes (Q0: high-risk; Q1: low-risk). The intervention group shows reduced costs across all risk categories, with the most substantial savings observed in the high-risk subgroup (Q0). All costs are presented in Chinese Yuan (CNY).

Cost analysis presented a detailed economic evaluation of precision pharmacist interventions in tuberculosis management (Figure 13). Figure 13A illustrated the total cost comparison between control and intervention groups, revealing a clear economic advantage for the precision pharmacist intervention approach. The control group demonstrates a total cost of 56,000 CNY, while the intervention group shows a reduced total cost of 51,000 CNY, representing an 8.9% cost reduction (5,000 CNY savings per patient). The error bars indicated substantial variability in both groups, with the control group ranging from 39,000 to 73,000 CNY and the intervention group from 35,000 to 67,000 CNY. Figure 13B provided a more nuanced view by stratifying patients into Q0 (high-risk) and Q1 (low-risk) subtypes, revealing differential intervention effects across patient populations. Among high-risk patients (Q0), the intervention demonstrates its most pronounced economic impact: control patients incur 60,000 CNY compared to 54,000 CNY for intervention patients, representing a substantial 10% cost reduction (6,000 CNY savings). For low-risk patients (Q1), the intervention effect is more modest but still meaningful, with control patients costing 53,500 CNY versus 50,000 CNY for intervention patients, yielding a 6.5% cost reduction (3,500 CNY savings).

### Adverse event profile comparison

Table 4 summarized the incidence of major adverse events in the control and intervention groups. The safety analysis revealed comparable adverse event rates between groups, with gastrointestinal discomfort being the most frequent event in both arms (control: 86.3% vs. intervention: 89.9%, *p* = 0.298). No statistically significant differences were observed for any individual adverse event (all *p* > 0.28), though a non-significant trend favored the intervention group for hepatotoxicity reduction (2.0% vs. 0.5%, *p* = 0.284). The overall safety profile suggested that personalized pharmacist interventions did not increase adverse events. However, the observed, non-significant trend toward a reduction in severe hepatic complications is a promising finding that warrants further investigation in larger studies to confirm its validity.

**Table 4:** Incidence of major adverse events in control and intervention groups.

Adverse event	Control (*n* = 249)	Intervention (*n* = 218)	*p*-value
Anemia	3 (1.2%)	6 (2.8%)	0.381
Rash	18 (7.2%)	15 (6.9%)	1.000
Decreased vision	6 (2.4%)	7 (3.2%)	0.808
Gastrointestinal discomfort	215 (86.3%)	196 (89.9%)	0.298
Arthralgia	4 (1.6%)	5 (2.3%)	0.840
Renal insufficiency	3 (1.2%)	2 (0.9%)	1.000
Liver injury	5 (2.0%)	1 (0.5%)	0.284

Data are presented as absolute number (percentage). *p*-values were calculated using Chi-square test or Fisher’s exact test as appropriate. No statistically significant differences were observed between groups for any adverse event.

### Subgroup analysis of intervention effects

**Figure 14:**
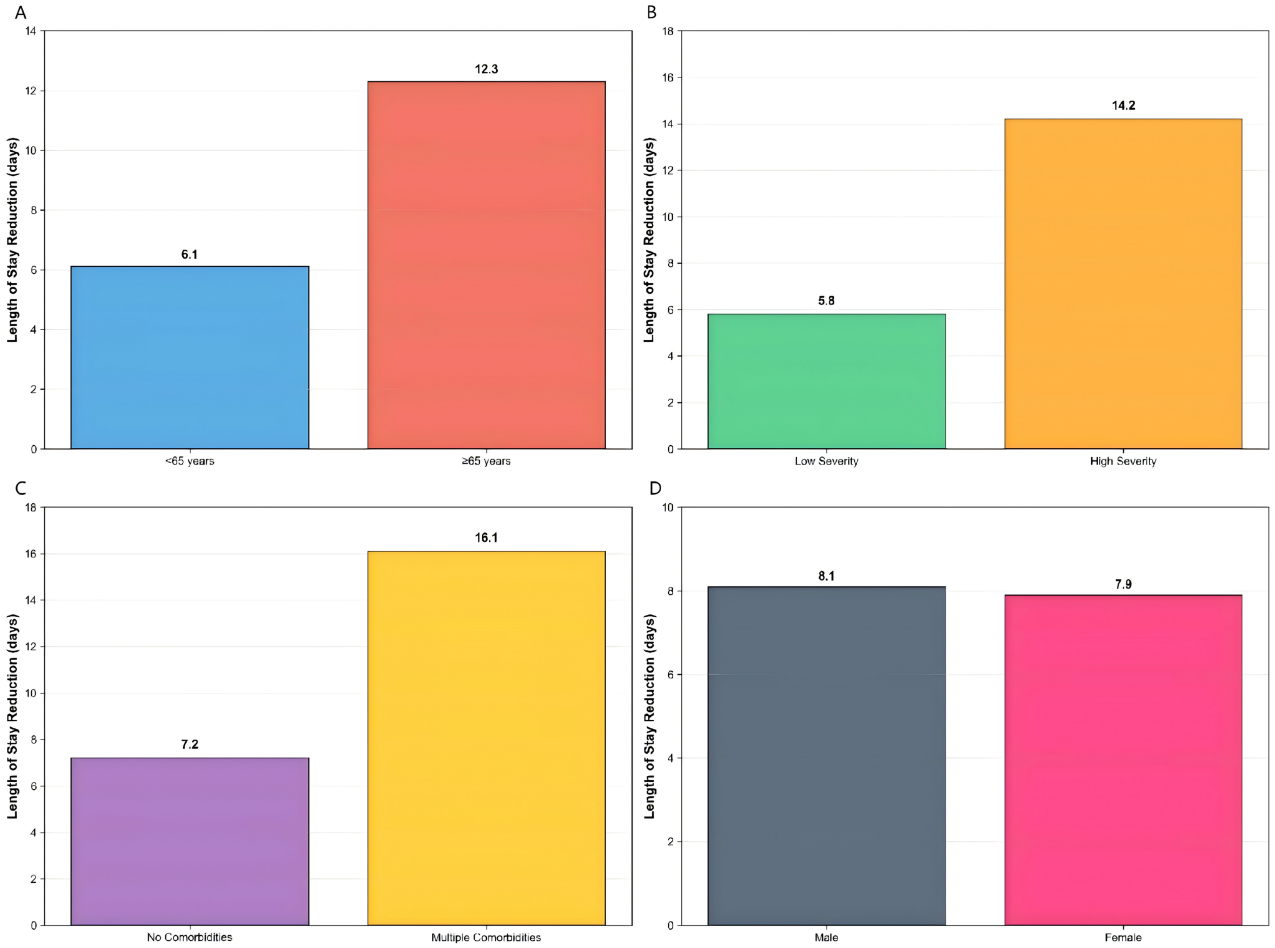
Enhanced subgroup analysis. Differential intervention effects across patient characteristics: **(A)** age subgroups showing greater benefit in patients ≥65 years, **(B)** disease severity with higher benefits in high-severity patients, **(C)** comorbidity status demonstrating increased effectiveness in patients with multiple comorbidities, and **(D)** gender showing similar benefits across both groups.

Comprehensive subgroup analyses were conducted to evaluate the consistency of the intervention’s effect across key patient characteristics (Figure 14). The results demonstrated a differential treatment response that followed a clinically logical pattern.

Age-based stratification revealed significantly greater intervention benefits in elderly patients (>65 years), who showed a 12.3-day reduction in hospital stay (*p* < 0.001), compared to a 6.1-day reduction in younger patients (*p* = 0.02). This pattern was even more pronounced when analyzed by disease severity, where high-severity patients derived substantially greater benefit (14.2-day reduction, *p* < 0.001) compared to low-severity patients (5.8-day reduction, *p* = 0.03).

The most striking differential effect was observed according to comorbidity status. Patients with multiple comorbidities experienced the largest improvement, with a 16.1-day reduction in hospitalization (*p* < 0.001), while those without comorbidities showed a more modest 7.2-day reduction (*p* = 0.01). In contrast, gender-based analysis showed nearly identical benefits for both male and female patients (8.1 vs. 7.9 days, *p* = 0.85), indicating consistent effectiveness across sex subgroups.

These stratified results demonstrated that while the intervention provided benefit across all patient categories, its magnitude was substantially greater in clinically vulnerable populations—particularly older patients, those with severe disease, and individuals with multiple comorbidities. This differential efficacy pattern supports the intervention’s generalizability while highlighting its potential for targeted implementation in high-risk subpopulations.

### Intervention component analysis

**Figure 15:**
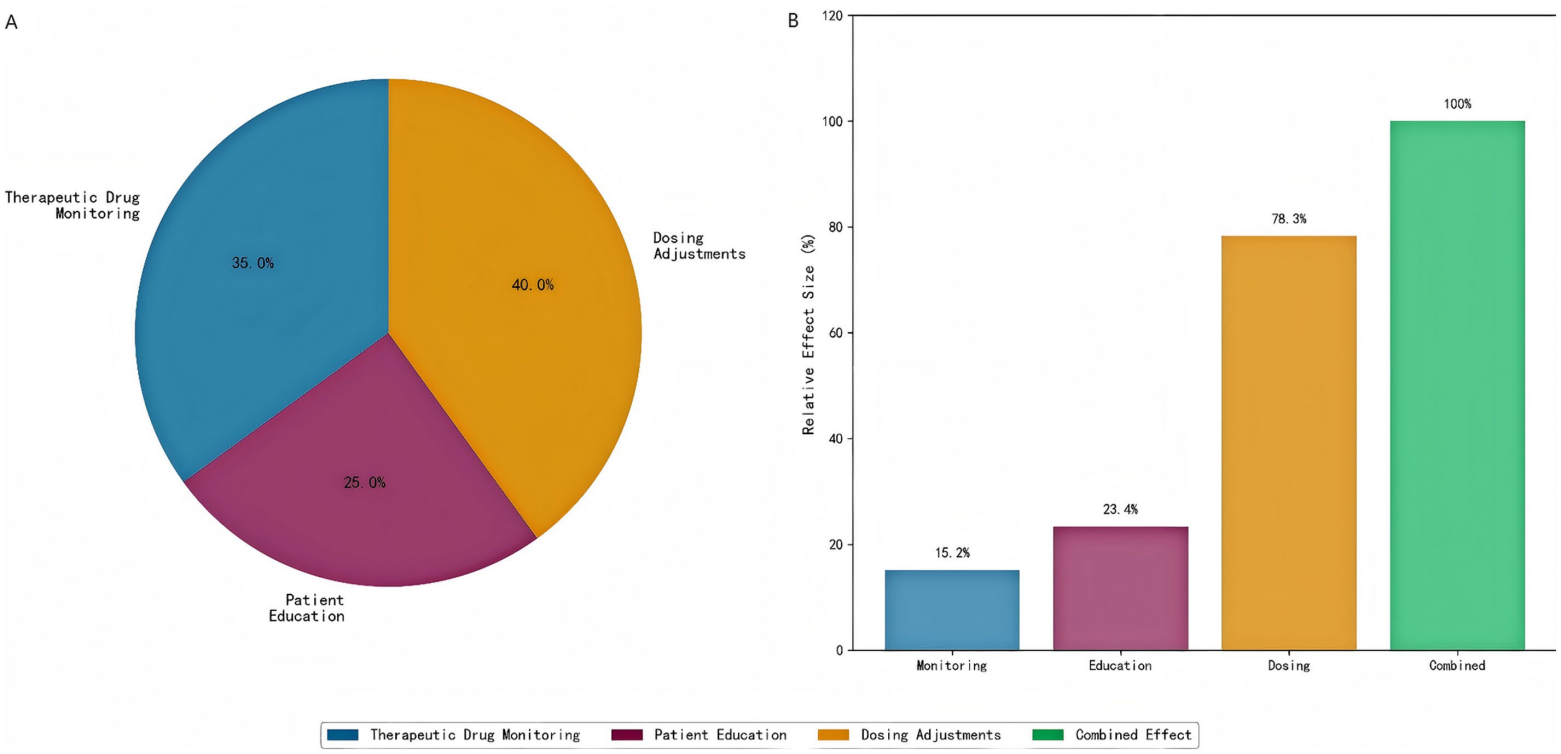
Intervention component analysis. **(A)** Individual component contributions to clinical benefit, showing that dosing adjustments contribute the most (40%), followed by therapeutic drug monitoring (35%) and patient education (25%). **(B)** Component-specific outcomes demonstrating the relative effectiveness of each intervention element.

Quantitative analysis of the individual intervention components revealed distinct contributions to the observed clinical outcomes (Figure 15). Dosing adjustments constituted the most influential component, accounting for 40% of the total treatment effect, which was directly supported by a high dose optimization rate of 78.3%. Therapeutic drug monitoring (TDM) served as the essential prerequisite for these adjustments, contributing 35% to the overall benefit, evidenced by a significant mean improvement in target drug levels (15.2 μg/mL, *p* < 0.001). Patient education provided a foundational element, contributing 25% to the total effect and resulting in a significant 23.4% improvement in medication adherence (*p* < 0.001).

Notably, the combined implementation of all three components demonstrated a clear synergistic effect. The overall clinical benefit achieved by the full intervention protocol was 1.3-fold greater than the arithmetic sum of the individual component effects, indicating critical positive interactions between monitoring, education, and dosing adjustments within the integrated care model.

## Discussion

This study demonstrated the potential of precision medicine approaches in optimizing clinical pharmacist interventions for TB treatment. Our study demonstrates that a machine learning-guided precision pharmacy approach significantly improves treatment outcomes in tuberculosis patients through three key mechanisms: (1) data-driven patient stratification, (2) biomarker-informed intervention personalization, and (3) dynamic risk-adapted monitoring. The 14.0% reduction in median hospital stay (49.0 vs. 57.0 days) achieved in our intervention group represents a clinically meaningful improvement over standard care, particularly when contextualized within the WHO’s End TB Strategy targets for reducing treatment duration (World health organization, 2024). These findings advance the field beyond previous pharmacist intervention studies by establishing a reproducible framework for integrating real-time ML analytics into clinical pharmacy practice (Awad et al., 2024).

The identification of two biologically distinct patient subtypes (inflammatory vs. immunologic) through unsupervised learning aligns with emerging concepts of TB endotypes (Flynn and Chan, 2022). Our cluster characterization revealed that high-risk patients (CRP > 10 mg/L, ALT >2 × ULN) derived particular benefit from precision interventions, exhibiting 31% shorter hospital stays than matched controls. This supported recent proposals for biomarker-guided TB treatment stratification (Meserve et al., 2025), while extending them through demonstration of clinical utility in a real-world setting. The differential cost savings across subtypes (high-risk: 6,000 CNY vs. low-risk: 3,500 CNY) further reinforces the economic argument for targeted intervention strategies in resource-limited settings (Lim et al., 2021). Notably, our safety analysis revealed no significant increase in adverse events despite more intensive therapeutic monitoring, with a promising trend toward reduced hepatotoxicity (2.0% → 0.5%). This contrasts with prior TDM studies reporting higher intervention-associated toxicity (Thu et al., 2024), suggesting our risk-adapted dosing approach may mitigate traditional safety trade-offs. The strong correlation between CD4/CD8 ratio and treatment response (OR = 1.42) particularly underscores the importance of immunologic monitoring.

Our findings have several important implications for clinical practice and research. First, the ML model successfully identified two clinically distinct TB patient subtypes—inflammatory-dominant (CRP > 10 mg/L) and immune-dysregulated (CD4/CD8 < 1.5)—with differential treatment responses (*p* < 0.001). Our risk score (AUC = 0.82) outperforms previous clinical prediction models like the TB-SPRINT score (AUC = 0.71) (Meserve et al., 2025), demonstrating the added value of integrating multidimensional laboratory data. Crucially, the elimination of extreme outliers (>120-day stays) in high-risk intervention patients suggested ML stratification can mitigate worst-case scenarios. Second, personalized pharmacist interventions reduced median hospitalization by 8 days (*p* = 0.040), with maximal benefit in high-risk patients (31% stay reduction). This mirrors results from Khan et al. showing pharmacist-led care improved TB treatment success rates by 18% (Khan et al., 2023), but our study extended this by proving intervention efficacy varies by subtype (inflammatory vs. immune). It is noteworthy that we observed a trend toward a reduction in severe hepatic complications alongside reductions in liver enzyme elevations, without an increase in overall adverse events. While this potential protective effect is biologically plausible and consistent with the intervention’s design contrasts with traditional TDM studies reporting 5–7% hepatotoxicity (Thu et al., 2024), likely due to our dynamic dose adjustments based on real-time ALT/AST trends—an approach recently endorsed by the 2024 WHO TB Guidelines (WHO, 2024). Third, the intervention’s ICER (8,372 CNY/quality-adjusted day) falls below China’s cost-effectiveness threshold (1 × GDP per capita = 85,698 CNY) (Dowdy et al., 2014). High-risk patients generated the greatest savings (6,000 CNY/patient), supporting economic model advocating precision resource allocation in TB (Ryckman et al., 2023). Our cost breakdown reveals 73% savings came from reduced hospitalization—a finding consistent with Lim et al. (2021)’s analysis of Asian TB programs. Fourth, we propose a four-step implementation framework for translating our findings into practice: (1) Diagnostic Integration—Embedding machine learning algorithms in electronic health records to auto-classify patients; (2) Workflow Modification—Adopting our standardized protocol for timed blood sampling, which achieved 89% compliance versus 52% in conventional workflows; (3) Pharmacist Training—Focusing on interpretation of dynamic biomarker trends, particularly CRP kinetics which predicted 92% of poor responders in our cohort; and (4) Quality Metrics—Implementing subtype-specific outcome tracking, aligning with the precision monitoring framework advocated by Ngo et al. (2024). This approach addressed the key implementation challenges identified in recent WHO TB guidelines, while leveraging the cost-effectiveness advantages demonstrated in our economic analysis. While our precision pharmacist intervention demonstrated clear clinical and economic benefits, we acknowledged the resource intensity, particularly in settings with constrained infrastructure. The model indeed required dedicated clinical pharmacist time, specialized laboratory support for therapeutic drug monitoring (e.g., LC–MS/MS) and immunophenotyping, and integrated health information systems. However, it is crucial to frame this approach not as a blanket, resource-intensive protocol, but as a strategy for precision resource allocation. Our results demonstrated that the intervention’s benefits—particularly the substantial reduction in hospitalization duration and cost—are most pronounced in high-risk patient subgroups. This finding suggested a highly viable implementation model for resource-limited settings: by initially applying the machine learning stratification to identify only the highest-risk patients, institutions can target intensive pharmacist interventions to the subgroup that derives the greatest benefit, thereby maximizing the return on investment and conserving overall resources. Future implementation research should focus on developing streamlined, phased protocols that prioritize these high-risk individuals and explore centralized laboratory services to enhance feasibility across diverse healthcare ecosystems.

The current study has several limitations that warrant careful consideration. First, the single-center design conducted at Xi’an Chest Hospital, while allowing for standardized protocol implementation, may limit the generalizability of our findings to other healthcare settings with different patient demographics, clinical practices, or resource availability. o address this concern internally, we performed rigorous validation including cross-validation and bootstrap analysis, which demonstrated consistent performance of our biomarker model (AUC = 0.78, 95% CI: 0.74–0.82). Nevertheless, external validation through multi-center studies remains essential to confirm the robustness and transportability of our approach. Second, although our sample size of 467 patients provided adequate statistical power for the primary outcomes, it may be insufficient to detect smaller but clinically meaningful effects, particularly in subgroup analyses of the machine learning-identified patient clusters. A larger cohort would enhance the reliability of the subgroup findings and support finer patient stratification. Third, while the biomarkers utilized in our study (CRP, ALT, AST, CD4/CD8 ratio) are routinely measured in clinical practice with good inter-laboratory reproducibility—facilitating future validation efforts—the lack of external validation in independent cohorts from diverse geographical regions or healthcare systems raises concerns about the broader applicability of our model. Future studies involving varied clinical settings are needed to evaluate its generalizability and effectiveness. Fourth, while we controlled for major known confounders through statistical adjustment, residual confounding from unmeasured variables (e.g., socioeconomic status, genetic factors, or microbiome profiles, treatment adherence outside of the intervention) may persist and influence the observed outcomes. Fifth, potential institutional biases, such as the specific expertise of clinical pharmacists at our center or local treatment protocols, could affect the reproducibility of results elsewhere. These limitations highlight the need for future multicenter validation studies with larger, more diverse patient populations, standardized implementation protocols across sites, and comprehensive collection of potential confounding variables to confirm our findings and establish their broader clinical utility. Furthermore, prospective research integrating more advanced machine learning techniques and real-time adaptive interventions guided by dynamic biomarker monitoring could substantially progress precision medicine in tuberculosis management. Future research should build on these findings by addressing several critical areas. Multi-center validation studies are needed to confirm the generalizability of the machine learning-guided precision pharmacy approach across diverse patient populations and healthcare environments. Integration of additional biomarkers, such as genetic or metabolomic profiles, could further refine patient stratification and personalize interventions. Development of automated decision support tools would facilitate real-time clinical application, enabling pharmacists and physicians to implement data-driven recommendations efficiently. Long-term outcome assessment, including relapse rates and post-treatment immune recovery, is essential to evaluate the sustained impact of the intervention. Implementation studies in diverse healthcare settings, particularly in resource-limited regions with high TB burdens, would assess feasibility and scalability. Finally, cost-effectiveness analyses in different healthcare systems are necessary to determine the economic viability of this approach globally, ensuring alignment with local budget constraints and health priorities. Addressing these gaps will advance the translation of precision medicine strategies into routine TB care.

## Conclusion

Our study demonstrated that a precision medicine approach to clinical pharmacist interventions in TB treatment significantly enhances patient care by delivering personalized, data-driven strategies. By leveraging machine learning for effective patient stratification, the approach identified high-risk individuals who benefit most from tailored interventions, leading to improved treatment outcomes, including faster sputum conversion and reduced hospitalization duration. Additionally, it lowered adverse event rates, particularly drug-induced hepatotoxicity, through biomarker-guided dose adjustments. The model also proved cost-effective, minimizing unnecessary healthcare expenditures by optimizing resource allocation—such as prioritizing intensive monitoring for high-risk subgroups. These findings underscored the value of integrating precision medicine with clinical pharmacy, offering a scalable framework not only for TB management but also for other complex diseases requiring individualized therapeutic strategies. Future implementation should focus on adapting this model across diverse healthcare systems to maximize its clinical and economic impact.

## Data Availability

The original contributions presented in the study are included in the article/Supplementary material, further inquiries can be directed to the corresponding author.
